# Synthetic Dermal Fillers in Treating Acne Scars: A Comparative Systematic Review

**DOI:** 10.1111/jocd.16752

**Published:** 2025-01-09

**Authors:** Salma Albargawi

**Affiliations:** ^1^ College of Medicine, Department of Dermatology Imam Mohammad Ibn Saud University Riyadh Saudi Arabia

**Keywords:** acne scars, CaHA, dermal fillers, HA, PMMA

## Abstract

**Background:**

Acne is a common condition observed in adolescents and in most severe acne the scars develop. There are numerous treatment options for acne scars. However, no standardized guidelines have been established to guide physicians in the optimal treatment of acne scars.

**Aims:**

The objective of this systematic review is to evaluate the existing evidence on various fillers used for the treatment of acne scars and to compare their effectiveness with one another.

**Methods:**

The study was designed following PRISMA guidelines, and the information was retrieved in May 2024 using the PubMed database and ClinicalTrials.gov registry. The inclusion criteria were that studies involving patients of any age or gender with acne scars of any type treated with synthetic dermal fillers, and studies published in English. The exclusion criteria were studies with less than 10 participants and studies that did not use synthetic dermal fillers. To assess the risk of bias in the included studies, the Cochrane Collaboration's Risk of Bias tool was used for randomized controlled trials, and in observational studies, the Newcastle–Ottawa Scale was used.

**Results:**

Twenty‐six studies were included with a total of 1121 participants. Fourteen studies evaluated HA on 372 subjects, five studies focused on PMMA on 305 subjects, four on CaHA on 392 subjects, two on PLLA on 42 subjects, and one on PCL on 10 subjects.

**Conclusions:**

Most of the studies included in this review were of low quality, as indicated by their scores on quality assessments, lack of high‐quality RCTs, and small sample sizes. Future research should focus on conducting randomized, controlled, split‐face studies with an adequate number of participants and a detailed examination of different scar subtypes.

## 1 Introduction

Scars form when the body's healing process produces either insufficient or excessive collagen. Acne is a common condition primarily affecting adolescents that is a common source of scarring [1]. Atrophic scars, which result from insufficient collagen, are the most common and include subtypes like ice pick, boxcar, and rolling scars. Ice pick scars, making up 60%–70% of atrophic scars, are deep, narrow indentations that penetrate the dermis. Boxcar scars are wide, rectangular depressions with sharp edges, while rolling scars appear as broad, undulating depressions [2]. In contrast, hypertrophic scars occur from excessive collagen production and are raised above the skin's surface. Keloid scars represent a more severe form of hypertrophic scarring. Among these, atrophic scars are more prevalent, with a ratio of 3:1 compared to keloid and hypertrophic scars [3].

Acne scars are permanent and can have significant psychological effects, making effective treatment essential. Treatment options are categorized as invasive and non‐invasive. Invasive methods, such as surgical procedures, offer quicker results and are typically used for severe or deep scars when non‐invasive treatments are insufficient. Non‐invasive treatments include topical treatments, chemical peels, laser therapy, microneedling, and dermal fillers [4]. This systematic review focuses on synthetic dermal fillers for treating acne scars.

### 1.1 Types of Dermal Fillers

Dermal fillers can be categorized as physical fillers (volume restoration) injected directly into the acne scars, filling in the depressions and raising the skin to the level of the surrounding tissue, such as hyaluronic acid (HA)‐based fillers. The other category is bio‐stimulatory fillers (collagen stimulators), which may add immediate volume while stimulating natural collagen production, such as poly‐l lactic acid (PLLA), polymethylmethacrylate (PMMA), calcium hydroxy apatite (CaHA), and polycaprolactone (PCL) fillers [5].

HA is a natural polymer used in dermal fillers, typically lasting 6–12 months and can be dissolved if needed, making them safe and versatile. HA fillers became widely popular due to their safety, efficacy, and the fact that they did not require allergy testing [6]. Most HA fillers, like Juvederm and Restylane, are cross‐linked for longer lasting results and are non‐animal derived, reducing allergy risks. PLLA filler (Sculptra) was initially approved for treating lipoatrophy in human immunodeficiency virus (HIV) patients [7]. In 2009, the FDA expanded its approval for correcting nasolabial folds and facial wrinkles. PLLA improves skin texture and volume by stimulating collagen production, providing gradual, long‐lasting improvement in facial volume and contour with results typically lasting around 2 years [8]. CaHA fillers, such as Radiesse, provide immediate volume while stimulating collagen, with results lasting 9–15 months [9]. They are known for their stability and safety. PMMA fillers, like Bellafill, offer long‐lasting results by supporting tissue and stimulating collagen production [10]. They are FDA approved for treating acne scars and provide long‐term results. PCL is a biodegradable and bioresorbable polymer [11]. Fillers like Ellansé and Gouri combine immediate volumizing effects with long‐term collagen stimulation [11]. These fillers are designed to improve skin texture and elasticity, offering durable results that last longer than many other fillers. Gouri is specifically marketed as a biostimulator, promoting natural collagen synthesis for a gradual and sustained improvement in skin quality.

This review is crucial in order to comprehensively evaluate and analyze the existing evidence, thereby providing clear guidance for clinical practice and enabling informed decision‐making for the clinician.

## 2 Methods

### 2.1 Study Design

This systematic review was conducted in accordance with the Preferred Reporting Items for Systematic Reviews and Meta‐Analyses (PRISMA) guidelines [12]. The objective was to evaluate the efficacy and safety of synthetic dermal fillers in the treatment of acne scars.

### 2.2 Ethics Statement

The author confirm that the ethical policies of the journal, as noted on the journal's author guidelines page, have been adhered to. No ethical approval was required as this is a review article with no original research data.

### 2.3 Eligibility Criteria

The review included studies involving patients of any age or gender with acne scars of any type treated with synthetic dermal fillers such as hyaluronic acid, calcium hydroxylapatite, poly‐L‐lactic acid, collagen, polymethylmethacrylate, and polycaprolactone. Studies that reported on treatment efficacy, patient satisfaction, adverse effects, or safety outcomes were considered.

Eligible study designs included clinical trials, cohort studies, case–control studies, case series, retrospective, and prospective studies, published in English. Studies that did not use synthetic dermal fillers, such as naturally sourced collagen or autologous fat, as intervention or treated unrelated conditions were excluded. Reviews, systematic reviews, and meta‐analyses, case reports, or studies with less than 10 participants were not considered; furthermore, non‐peer‐reviewed articles such as abstracts, editorials, letters, and opinion pieces were excluded.

### 2.4 Search Strategy and Study Selection

A comprehensive literature search was performed in May 2024 using the PubMed database and ClinicalTrials.gov registry to identify relevant studies. The search was filtered to papers published from 2004 to 2024. The search terms included combinations of the following keywords: “Dermal Fillers,” “Acne Scars,” “Atrophic Scars,” “Facial Scars,” “Efficacy,” “Safety”. Boolean operators were used to combine these terms, ensuring a comprehensive search. Titles and abstracts retrieved from the initial search were screened. Full‐text articles of potentially eligible studies were then assessed based on the inclusion and exclusion criteria.

The following data were extracted from the included articles: author names, year published, title, journal, country, study design, sample size, demographics, type of filler, conjunctive treatment (if any), description of intervention (technique, volume injected, follow‐up), comparison group, outcome measures, measures used, main results, and side effects.

### 2.5 Risk of Bias Assessment

The risk of bias in included studies was assessed using appropriate tools based on the study design. For the 12 randomized controlled trials (RCTs), Cochrane's Risk of Bias 2.0 tool was employed, while the NHLBI Quality Assessment Tool for Case Series Studies was applied to the 3 case series. Additionally, Cochrane's ROBINS‐I tool (Risk Of Bias In Non‐randomized Studies—of Interventions) was used for the remaining studies.

## 3 Results

**FIGURE 1:**
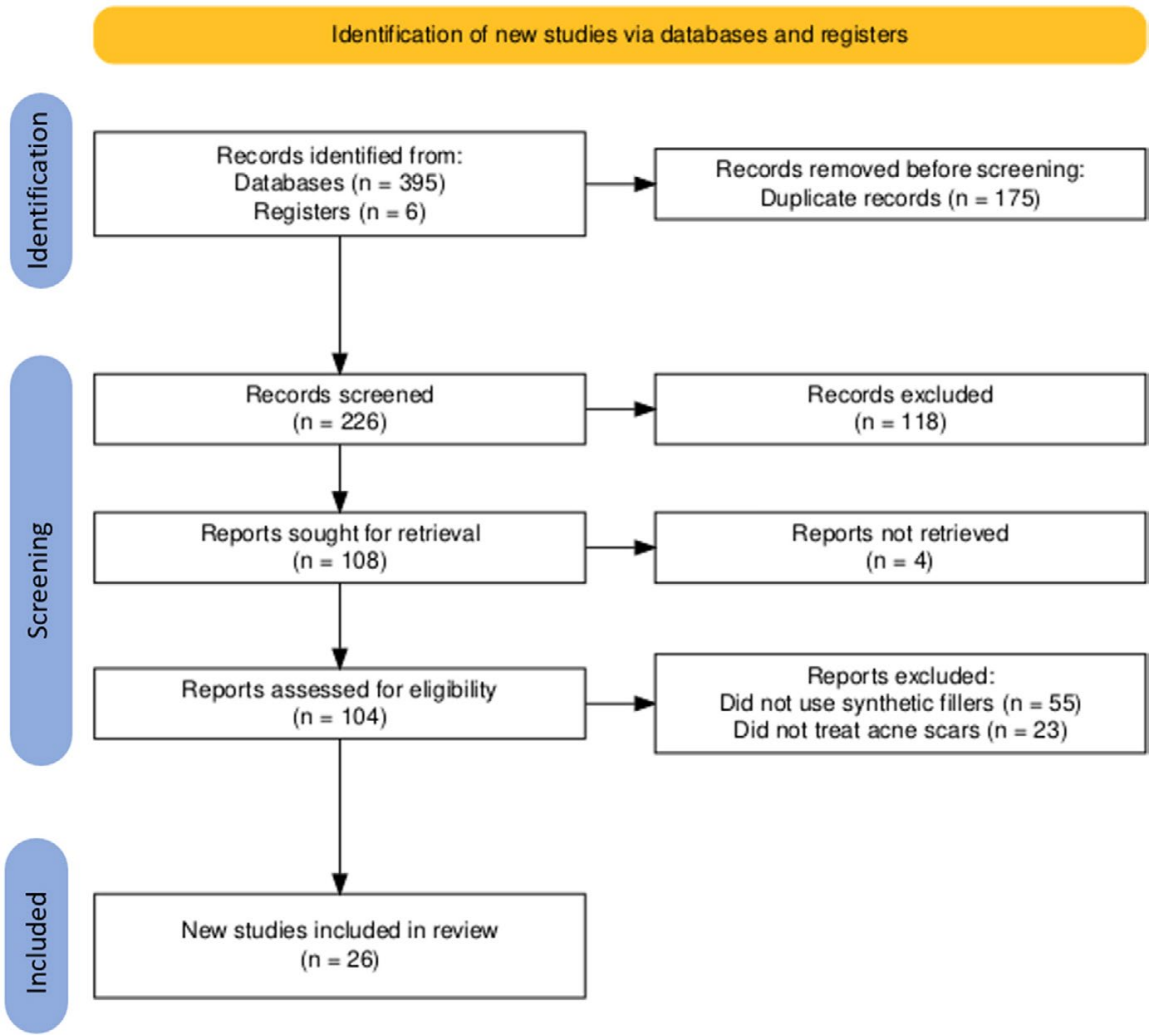
PRISMA flowchart. Preferred reporting items for systematic reviews and meta‐analyses.

**FIGURE 2:**
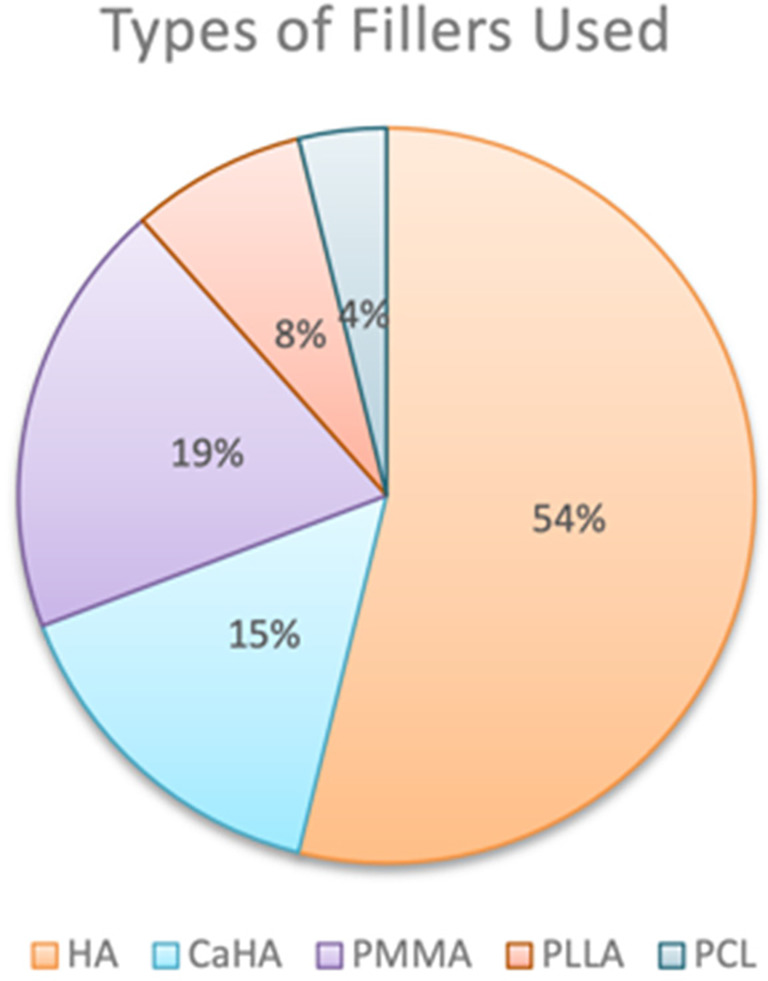
Percentage of types of fillers used in the included studies. CaHA, calcium hydroxy apatite; HA, hyaluronic acid; PCL, polycaprolactone; PLLA, poly‐l lactic acid; PMMA, polymethylmethacrylate.

The initial search yielded 401 results, with 175 duplicates removed. After applying eligibility criteria to the screened abstracts, 118 studies were excluded. An additional 82 were removed due to irretrievability and failure to meet the inclusion and exclusion criteria (Figure 1). Ultimately, 26 studies were included in the final review (Table 1). Among the 26 studies, 14 evaluated HA, 5 focused on PMMA, 4 on CaHA, 2 on PLLA, and 1 on PCL (Figure 2). The studies included 12 randomized trials (4 double‐blind, 7 single‐blind, and 1 open‐label), 1 nonrandomized trial, 5 single‐arm studies, 5 retrospective analyses, and 3 case series.

**TABLE 1:** Two of the case series studies were rated as fair quality, and one was rated as good quality.

Quality assessment tool for case series studies											
Authors	1	2	3	4	5	6	7	8	9	Grade	%
Artzi et al. [13]	1	1	1	1	1	0	0	0	0	Fair	55.5%
Halachmi et al. [48]	1	1	1	1	1	1	1	0	1	Good	88.8%
Belmontesi [14]	1	1	1	1	1	1	1	0	0	Fair	77.7%

*Note:* 1: Yes; 0.5: CD; 0: No (Good > = 80%, Fair > = 50%, Poor < 50%).

### 3.1 Quality Assessment

**FIGURE 3:**
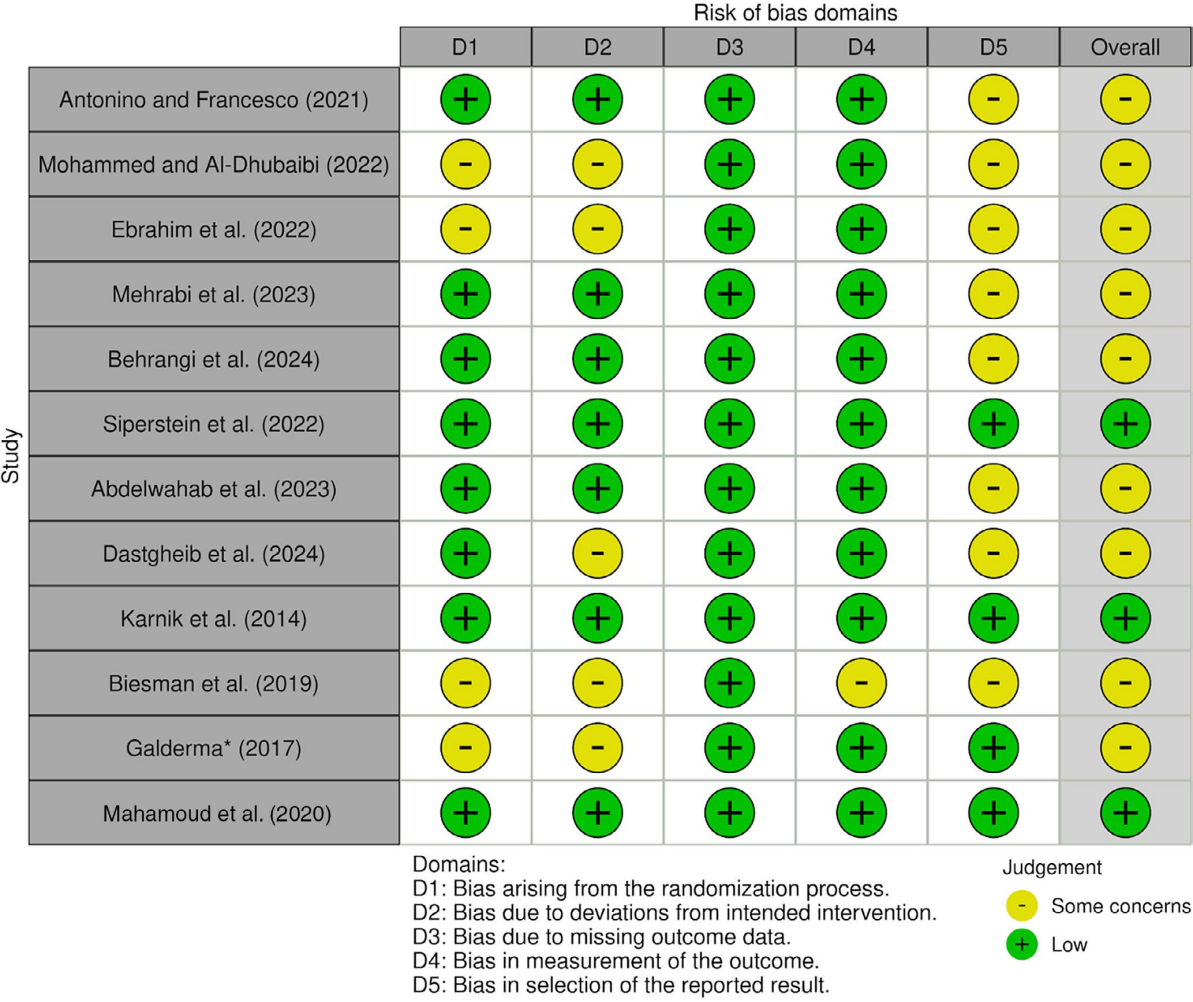
Aricles assessed with ROB‐2. Nine showed some concerns and the remaining three presented a low risk.

In assessing the risk of bias for the included studies, we employed Cochrane's Risk of Bias 2.0 tool for the 12 RCTs. Our analysis, depicted in Figure 3, indicates that 9 (75%) of the 12 studies raised some concerns, highlighted in yellow, while the remaining 3 (25%) studies were assessed as having a low risk of bias, marked in green. This assessment revealed that the majority of the selected RCTs fell into the “some concern” category, with only a few demonstrating a low risk of bias.

**FIGURE 4:**
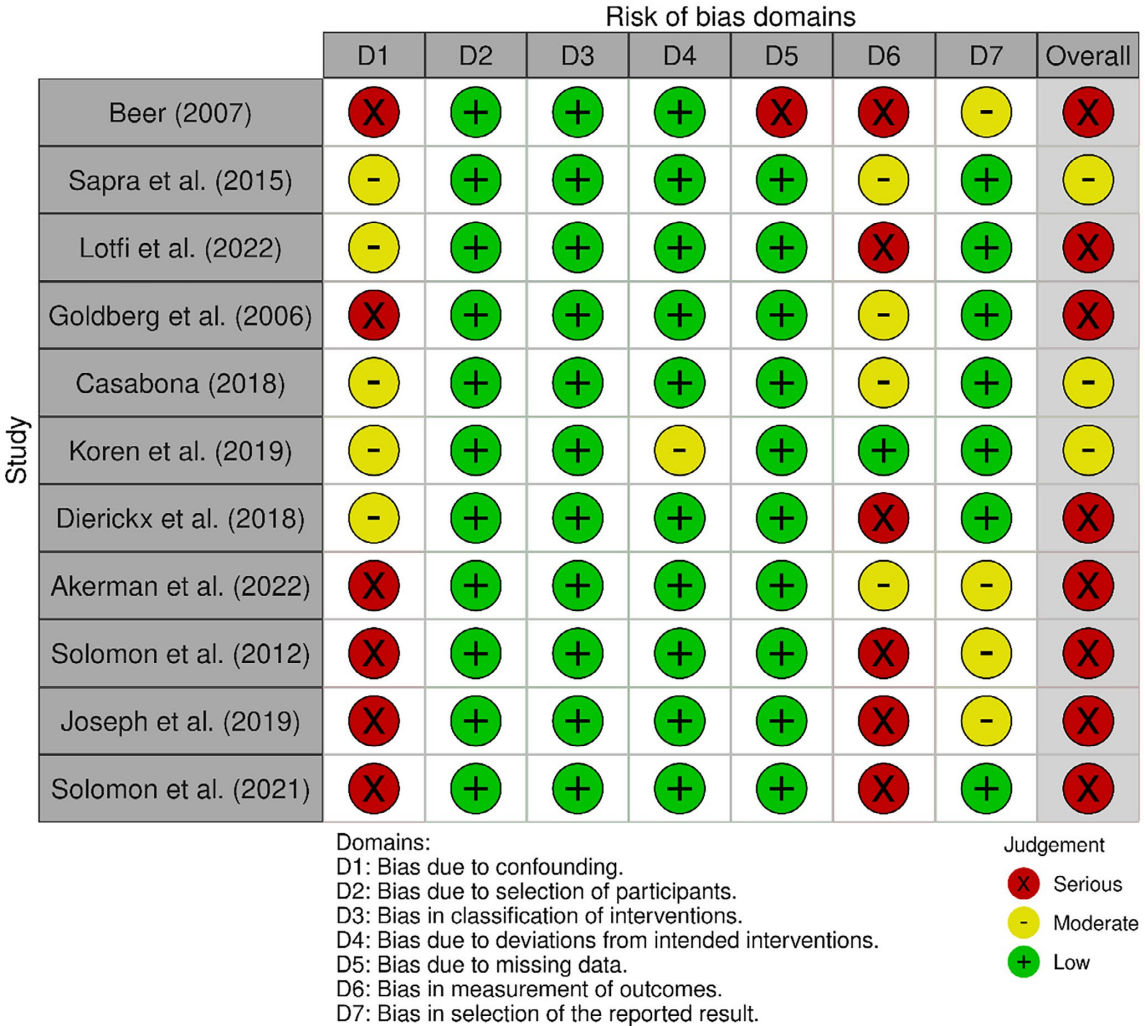
Articles assessed with ROBIN‐I tool. Eight showed serious risk and three showed moderate.

For the non‐randomized studies, Cochrane's ROBINS‐I tool was used, and the results are shown in Figure 4. Of the 11 studies evaluated, 8 (73%) presented a serious risk of bias, highlighted in red, while the remaining 3 (27%) studies were categorized as having a moderate risk of bias, marked in yellow.

As for case series studies shown in Table 1, two studies were assessed as fair quality based on the checklist items met. As well as, one case series was classified as good quality.

Table 2 summarizes the characteristics of the included studies and Table 3 summarizes the main results.

**TABLE 2:** Characteristics of included studies.

Authors	Country	Study design	No. of patients	Participant demographics	Type of dermal filler	Combination treatment
Koren et al. [15]	Israel	Retrospective	352	Mean age: 28.7 ± 8.7, 231 female; 121 male; Fitzpatrick II: 30.7%, Fitzpatrick III: 39.8%, and Fitzpatric IV: 29.5%; treated with FACL, RF, NAFL, and CaHA	CaHA (Radiesse)	FACL, RF, NAFL
Casabona [16]	Brazil	Retrospective	10	Mean age: 37.6 ± 10.0 years; at least one prior treatment for their atrophic acne scars	CaHA (Radiesse)	MFU‐V; Ultherapy
Goldberg, Amin, and Hussain [17]	USA	Single‐blind, Single‐arm	10	Aged between 16 and 60; at least one saucerized acne‐induced scar	CaHA (Radiesse)	Monotherapy
Antonino and Francesco [18]	Italy	Double‐blind, randomized controlled	20	Aged between 30 and 50 years; all women; moderate‐to‐severe atrophic scars	CaHA (Radiesse)	Monotherapy at 1 month, followed by HIFU
Mehrabi et al. [19]	Iran	Double‐blind, randomized controlled	30	Mean age: 36.6 ± 10.9 years; 27 females and 3 males; Fitzpatrick I: 1, II 16, and III 13; moderate‐to‐severe atrophic acne scars on both cheeks	CL‐HA (Juvéderm Voluma) and NCL‐HA (Profhilo)	Subcision
Behrangi et al. [20]	Iran	Double‐blind, randomized controlled	16	Aged between 18 and 40 years; all females; with atrophic acne scars	CL‐HA (Neuvia Intense Rheology) and NCL‐HA (Revitacare Cytocare)	Botulinum Toxin A
Siperstein et al. [21]	USA	Single‐blind, randomized controlled	15	Aged between 26 and 59; 11 females and 4 males; at least two of each Fitzpatrick skin type	HA (Juvéderm Volift)	Monotherapy
Mohammed and Al‐Dhubaibi [22]	Egypt	Single‐blind, randomized controlled	82	Mean age: 24.4 ± 2.3 years (experimental) and 26.1 ± 1.2 years (control); 53 females and 29 males; atrophic acne scarring	HA (Profhilo)	Subcision
Dastgheib et al. [23]	Iran	Single‐blind, randomized controlled	12	Mean age: 29.75 years (19–42 years); 9 females and 3 males; Fitzpatrick skin type II–IV; atrophic acne scars on both sides of the face	HA (Profhilo)	Subcision
Akerman et al. [24]	Israel	Retrospective	12	Mean age: 44 ± 5.2; 11 females and 1 male; Fitzpatrick I–III; mild to moderate post acne scars	HA (Profhilo)	NAFL
Artzi et al. [13]	Israel	Case series	12	Mean age: 33.2 ± 9 years; Fitzpatrick skin phototypes II–IV and moderate‐to‐severe atrophic acne scars	HA (Profhilo)	Subcision
Dierickx, Larsson, and Blomster [25]	Belgium	Open‐label, single‐arm	12	Mean age: 33 years (27–43 years); 8 females and 4 males; Caucasians with moderate to severe acne scars	HA (Restylane Vital Lidocaine)	Monotherapy
Halachmi et al. [48]	Israel	Case series	12	Aged between 19 and 54 years; 5 females and 7 males; moderate to severe acne scarring who had completed fractional laser resurfacing	HA (Restylane Vital Lidocaine)	FACL or Fractional Er: YAG
Galderma ^a^ [50]	Germany	Single‐blind, randomized controlled	49	Mean age: 40.3 ± 9.9 years; 10 males and 39 females; Fitzpatrick I–IV	HA (Restylane Vital Lidocaine)	Monotherapy
Belmontesi [14]	Italy	Case series	10	Aged between 19 and 25 years; 7 females and 2 males; moderate to severe acne, presenting with atrophic scars	HA (Restylane)	Topical retinoid
Ebrahim et al. [26]	Egypt	Single‐blind, randomized controlled	40	Mean age: 26.65 ± 6.77 years; 22 females and 18 males; moderate‐to‐severe acne scars; Mean duration of acne scars: 3.78 ± 2.15 years	HA (STYLAGE XL)	Subcision
Abdelwahab, Omar, and Hamdino [27]	Egypt	Single‐blind, randomized controlled	40	Mean age: 27.93 ± 6.05 years; 19 females and 21 males; Mean duration of acne scars: 5.5 (3.5–7) years	HA (STYLAGE XL)	Subcision
Mahamoud et al. [49]	Egypt	Single‐blind, randomized controlled	30	Mean age: 30.1 ± 5.49; 16 females and 13 males; Fitzpatrick III: 19, IV: 8, V: 3; Mean duration of scars: 10.18 ± 5.38 years	HA (Viscoderm 24 mg/mL)	FACL
Sapra et al. [28]	Canada	Open‐label, single‐arm	22	Mean age: 37.6 6 ± 9.17 years; Rolling scars	PLLA (Sculptra)	Monotherapy
Beer [29]	USA	Open‐label, single‐arm	20	Mean age: 42.4 7 ± 10.7 years; 10 males, 10 females; facial scars from moderate to severe acne or varicella	PLLA (Sculptra)	Monotherapy
Solomon, Sklar, and Zener [30]	Canada	Retrospective	24	Not stated; depressed acne scars	PMMA (Artecoll)	Monotherapy
Karnik et al. [31]	USA	Double‐blind, randomized controlled	147	Mean age: 44 years, 61% female and 39% male	PMMA (Artefill)	Monotherapy
Joseph et al. [10]	USA	Open‐label, nonrandomized	42	Mean age: 43; 62% female and 38% male; Fitzpatrick I–III: 45% and Fitzpatric IV–VI: 55%; distensible atrophic acne scars	PMMA (Bellafill)	Monotherapy
Solomon et al. [32]	Canada	Retrospective	48	Not stated; depressed acne scars	PMMA (Bellafill)	Monotherapy
Biesman et al. [33]	USA	Open‐label, randomized	44	Media age: 34 years (23–67 years); 15 men and 29 women; Fitzpatrick I–IV; at least 4 distensible facial acne scars	PMMA (Bellafill)	Microneedling
Lotfi, Shafie'ei, and Ahramiyanpour [34]	Iran	Open‐label, single‐arm	10	Mean age: 31.3 ± 0.83 years; 3 males and 7 females; Fitzpatric III: 6 and Fitzpatric IV: 4; moderate to severe mixed atrophic facial acne scarring	Polycaprolactone (Ellansé)	Subcision

Abbreviations: CaHA, calcium hydroxyapatite; CL, cross‐linked; FAC, fractional CO_2_ laser; HA, hyaluronic acid; HIFU, high‐intensity focused ultrasound; MFU‐V, micro focused ultrasound with visualization; NAFL, non‐ablative laser; NCL, non‐cross‐linked; PCL, polycaprolactone; PLLA, poly‐L lactic acid; PMMA, polymethylmethacrylate.

^a^ Conducted by Galderma, Clinicaltrials.gov registry did not list a primary investigator.

**TABLE 3:** Results of included studies.

Authors	Type of dermal filler	Combination treatment	Results	Adverse effects
Goldberg, Amin, and Hussain [17]	CaHA (Radiesse)	Monotherapy	At the 12‐month follow‐up, 3 subjects showed 75% improvement; 6 showed between 50% and 75% improvement, and 1 subject showed between 25% and 50% improvement in their saucerized acne scars. No subjects showed any improvement in their ice‐pick scars	Ecchymosis, edema, erosion, erythema, extrusion, hematoma, infection, and nodule and further scar formation
Antonino and Francesco [18]	CaHA (Radiesse)	Monotherapy at 1 month, followed by HIFU	CaHA significantly improved wrinkles, skin texture, and atrophic acne scars at 1 month (*p* < 0.05). HIFU alone improved wrinkles, skin texture, and hemoglobin at 3 and 6 months, but combining CaHA with HIFU showed no additional benefit over HIFU alone	None reported
Koren et al. [15]	CaHA (Radiesse)	FACL, RF, NAFL	FACL demonstrated significantly higher GAS scores than CaHA alone (*p* ≤ 0.001). No significant differences were found between RF and diluted CaHA scores. Combining FACL and CaHA yielded significantly higher GAS scores than FACL alone (*p* ≤ 0.001). CaHA‐based filler combined with EBDs increased treatment efficacy, whether in same‐session or separate‐session treatments (*p* = 0.23). CaHA treatment showed better results than NAFL (*p* < 0.02) and combining CaHA with EBD produced superior results compared to EBD alone. Patient demographics and scar characteristics did not significantly influence outcomes. FACL achieved superior results with fewer sessions compared to NAFL and RF (*p* < 0.001)	CaHA and EBD led to more Aes than EBD alone
Casabona [16]	CaHA (Radiesse)	MFU‐V; Ultherapy	Significant overall improvement in baseline acne scar severity at day 90. Overall mean acne scar scores decreased from 7.6 ± 1.7 before treatment to 3.8 ± 0.9 (*p*‐value = 0.002) at day 90. Clinical improvements did not achieve statistical significance. Subjects with mild scars decreased mean acne scores from 1.0 ± 0.5 to 0.7 ± 0.5 (*p*‐value = 0.250) while scores decreased from 2.8 ± 1.8 to 1.1 ± 1.5 (*p*‐value = 0.063) among subjects with severe scars. A single subject with a hyperplastic popular scar also achieved clinical improvement. GASS scores were significant for improvement in total acne scars, but not for mild, moderate, or hyperplastic popular scars	None reported
Mehrabi et al. [19]	CL‐HA (Juvéderm Voluma) and NCL‐HA (Profhilo)	Subcision	Both JV and *p* fillers showed significant acne scar volume improvement at V3 (9% and 6%, respectively) compared to baseline. *p* showed further improvement at V4 (16%), while JV did not (10%). JV's Iex reduced significantly from visit 1 to visit 3 (*p* < 0.001) but remained unchanged afterward. *p* showed a significant Iex reduction between visits 3 and 4 (*p* < 0.001). The hemiface treated with *p* had a greater reduction in *R* ^2^ compared to JV (*p* < 0.01). IGA scores improved most for JV between visits 1 and 3 (*p* < 0.01) and for *p* between visits 3 and 4 (*p* < 0.05). Patient perceptions aligned with these findings, though not statistically significant	Edema, erythema, and scabbing of the injected area
Behrangi et al. [20]	CL‐HA (Neuvia Intense Rheology) and NCL‐HA (Revitacare Cytocare)	Botulinum Toxin A	At the first follow‐up, no significant differences were found between cross‐linked and non‐cross‐linked HA in fine pores, large pores, or acne severity. By the second follow‐up, cross‐linked HA had a significantly higher number and area of large pores (*p* = 0.01). Both groups saw significant reductions in fine pores from the first to the last session (*p* = 0.001), though changes between groups were not significant (*p* = 0.06). Acne severity improved significantly in both groups, with severe cases reduced to 0% by the final session	None reported
Siperstein et al. [21]	HA (Juvéderm Volift)	Monotherapy	The HA filler group showed a significantly greater reduction in QGSGS scores (−6.6) compared to the saline group (−1.7) [t (28) = −4.320, *p* = 0.0008], with similar findings from the investigator (−7.1 vs. −1.4) [t (28) = −5.043, *p* = 0.0002]. The number of rolling atrophic scars also significantly decreased in the HA filler group (−8.1 vs. −2.1) [t (28) = −6.283, *p* = 0.00003], confirmed by both the blind evaluator and investigator. Saline alone also showed a smaller but significant reduction in QGSGS scores and atrophic scars. Ninety days post‐treatment, 93% of subjects preferred the HA filler, with 93% reporting improvement on GAIS and 71% stating improvement much or very much	Bumps on Day 1
Mohammed and Al‐Dhubaibi [22]	HA (Profhilo)	Subcision	Clinical improvement was achieved in both groups, with significant improvements in the acne scar severity index, Goodman and Baron grading scale, and patient satisfaction (*p* < 0.05). The triple step technique group showed significantly greater improvement than the subcision group in these measures (*p* < 0.05). Rolling and boxcar scars improved significantly more than icepick scars in both groups (*p* < 0.05). The study group showed greater “mean improvement” and “70% improvement or more” compared to the control group, with patients reporting a noticeable reduction in scar depth and size and better skin appearance	None reported
Akerman et al. [24]	HA (Profhilo)	NAFL	Improvement in overall scar appearance was gradual, with all patients showing a 25%–50% improvement by the end of treatment. Evaluator 1 reported a mean improvement of 1.63 points (SD = 0.5), and evaluator 2 reported 2.13 points (SD = 0.65), with an overall mean of 1.88 points (SD = 0.57). Interobserver agreement was 80%. Patient satisfaction at 3 months ranged from 3 to 5, with a mean of 4 (SD = 1.06)	Erythema, edema, pain
Dastgheib et al. [23]	HA (Profhilo)	Subcision	The total and boxcar scores were significantly higher on the HA filler + subcision side compared to subcision alone, while icepick and rolling scores were not. Patient satisfaction VAS scores increased more on the HA filler + subcision side (528.08%) than on the subcision side (219.06%) (*p* = 0.021). The mean depth reduction was similar between both methods, with no significant difference in the growth rate of sonographic depth or total score. The rolling subtype responded better on the HA filler + subcision side, while boxcar scores improved more on the subcision side. No significant difference was observed in the reduction percentage of clinical scores between both sides for other subtypes	Erythema, swelling, ecchymosis, significant pain, hyperpigmentation
Artzi et al. [13]	HA (Profhilo)	Subcision	Eight out of 12 patients reported moderate improvement, 2 marked improvement, and 2 minimal improvements. Dermatologists' mean global evaluation score at 6 months was 2.5 ± 0.43	Minor visible HA deposits in two patients, which disappeared with manual pressure
Galderma ^a^ [50]	HA (Restylane Vital Lidocaine)	Monotherapy	Significant improvement in scar severity was noted in the treated cheek by blind evaluator and by GAIS measure	None reported
Dierickx, Larsson, and Blomster [25]	HA (Restylane Vital Lidocaine)	Monotherapy	67% of subjects improved on SCAR‐S at Week 36. 92% of subjects were assessed as improved on GAIS by investigator at Week 36	Nodules, erythema
Halachmi et al. [48]	HA (Restylane Vital Lidocaine)	FACL or Fractional Er: YAG	All the patients with ice pick acne scars underwent HA filler microinjection after a series of fractional resurfacing treatments. Procedure was well‐tolerated, and all patients were extremely satisfied with the immediate improvement	Transient pinpoint bleeding at injection site
Belmontesi [14]	HA (Restylane)	Topical retinoid	Sequential treatment with topical trifarotene and injectable HA gel is effective in improving the appearance of atrophic acne scars, with high rates of clinical improvement and patient adherence	None reported
Ebrahim et al. [26]	HA (STYLAGE XL)	Subcision	Two months post‐treatment, both the subcision + HA (76.5% grade I) and subcision + threads groups (70.6% grade I) showed significant improvement over subcision alone (*p* < 0.0001), with no significant difference between them. Subcision alone saw 41.2% reach grade II (*p* = 0.0001). Physician assessments rated 76.5% of the HA group and 70.6% of the threads group as excellent (*p* < 0.0001), with higher patient satisfaction in the combination groups (*p* < 0.0001). The best results were for rolling/moderate acne scars (*p* = 0.74)	Pain, edema, ecchymosis
Abdelwahab, Omar, and Hamdino [27]	HA (STYLAGE XL)	Subcision	At 6 months, the HA filler and fractional CO_2_ laser sides showed significantly better outcomes than subcision (*p* = 0.015 and *p* < 0.001, respectively), with no significant difference between HA filler and fractional CO_2_ laser (*p* = 0.171). Both qualitative and quantitative Goodman and Baron scores were significantly better in the HA filler and fractional CO_2_ laser groups compared to subcision (*p* < 0.001). Patient satisfaction was also higher in these groups (*p* = 0.011, < 0.001)	Pain, erythema, edema (lasting 3–10 days in most patients)
Mahamoud et al. [49]	HA (Viscoderm 24 mg/mL)	FACL	Qualitative Goodman and Baron scores showed no significant difference between PRP and HA at baseline (*p* = 0.959) or post‐treatment (*p* = 0.921). Both treatments significantly reduced scar severity (*p* < 0.001). Quantitative scores improved by 33% for PRP (13.39 ± 4.11 to 8.97 ± 4.34, *p* < 0.001) and 21.72% for HA (13.07 ± 3.95 to 10.23 ± 3.86, *p* < 0.001), with PRP showing better results. Patient feedback showed no significant difference, with most reporting marked improvement	None reported
Beer [29]	PLLA (Sculptra)	Monotherapy	Significant reductions in scar severity were observed over 7 treatments, with a maximum reduction of 46.4% by the seventh session. While cumulative reductions increased with continued treatments, changes were not significant after reducing injected volume during the six follow‐up sessions. Subject ratings showed significant reductions in severity across the 7 treatments, and satisfaction scores trended upward, though not significantly	None reported
Sapra et al. [28]	PLLA (Sculptra)	Monotherapy	Progressive improvement was observed in mean PSIS and BESIS scores, decreasing from session 2 (3.4–3.9 and 3.7–3.9) to the follow‐up visit (2.4–2.9 and 2.2–2.5). The highest proportion of patients achieving a PSIS and BESIS score of ≤ 2 at follow‐up was seen with the VISIA‐CR camera system (63.6% and 68.2%). Similarly, SASIS scores decreased from session 2 (3.4 and 1.4) to follow‐up (2.4 and 2.5), with the highest proportion of patients achieving a SASIS score of ≤ 2 and an STSS score of ≥ 3 at follow‐up using VISIA‐CR images (45.5% and 54.5%)	Palpable nonvisible nodule, acne, and post inflammatory pigmentation
Solomon, Sklar, and Zener [30]	PMMA (Artecoll)	Monotherapy	95.8% noted significant subjective improvement of their acne scars, 37.5% noticed a palpable textural change to their skin from the injection, and none had significant granuloma formation or visible lumpiness	Echymosis, erythema, and itching
Karnik et al. [31]	PMMA (Artefill)	Monotherapy	Statistically significant favorable outcomes in the efficacy of PMMA‐collagen as measured by ASRS, PGAIS, and SGAIS	Localized pain, bruising, swelling and acne.
Biesman et al. [33]	PMMA (Bellafill)	Microneedling and subcision	The combination of microneedling and subcision with PMMA‐collagen gel injections demonstrated significant effectiveness for the treatment of facial acne scars for at least 36 weeks as assessed by ASAS, PGAIS, and SGAIS scores	None reported
Joseph et al. [10]	PMMA (Bellafill)	Monotherapy	At 4 and 7 months after initial treatment, ≥ 1‐point improvement on the ASAS measure. Extremely elevated levels of aesthetic improvement were noted by both the physicians and subjects on the GAIS. QOLIS measure showed that 30% of subjects had at least a 1‐point improvement or better after treatment	Bruising and ecchymosis
Solomon et al. [32]	PMMA (Bellafill)	Monotherapy	99% of patients reported improvement and were satisfied	Nodules, edema
Lotfi, Shafie'ei, and Ahramiyanpour [34]	Polycaprolactone (Ellansé)	Subcision	The total number of acne lesions (post‐intervention range: 3–15) decreased by 11.90 ± 5.53 (*p*‐value of < 0.001). The total number of ice peak lesions (post‐intervention range: 0–12) decreased by 5.70 ± 4.24 (*p*‐value = 0.002), rolling lesions (post‐intervention range: 1–3) decreased by 5.20 ± 2.25 (*p*‐value < 0.001), and boxcar lesions (post‐intervention range: 0–3, with 30% of individuals having zero number of the lesion and 80% overall having one or less) decreased by 1 ± 0.94 (*p*‐value = 0.023). Statistically significant favorable responses to the qualitative and quantitative grading of scars (*p*‐values of 0.004 and < 0.001, respectively), and all individuals reported improvement	Edema, ecchymosis, burning, and hypoesthesia

Abbreviations: CaHA, calcium hydroxyapatite; CL, cross‐linked; FAC, fractional CO_2_ laser; HA, hyaluronic acid; HIFU, high‐intensity focused ultrasound; MFU‐V, micro focused ultrasound with visualization; NAFL, non‐ablative laser; NCL, non‐cross‐linked; PCL, polycaprolactone; PLLA, poly‐L lactic acid; PMMA, polymethylmethacrylate.

^a^ Conducted by Galderma, Clinicaltrials.gov registry did not list a primary investigator.

## 4 Discussion

Acne scars are permanent skin changes that result from acne vulgaris. Managing acne scars remains a significant challenge for clinicians due to the varying efficacy of available treatment options. Although the literature on the prevalence and epidemiology of acne scars is limited, it is estimated that some degree of scarring occurs in up to 95% of individuals with acne, with around half presenting with clinically significant scars [35, 36]. Current treatment options include lasers, chemical peels, dermal fillers, and subcision, among others [37]; however, no standardized guidelines have been established to guide physicians in the optimal treatment of acne scars. The objective of this systematic review is to evaluate the existing evidence on various fillers used for the treatment of acne scars and to compare their effectiveness with one another.

### 4.1 Hyaluronic Acid Filler as Monotherapy

We identified three controlled studies that explored the use of HA filler as a monotherapy for the treatment of acne scarring. The first study, an open‐label trial involving 12 Caucasian subjects with moderate to severe acne scars, reported improvements in scarring, as measured by the Global Aesthetic Improvement Scale (GAIS) and Scale for Acne Scar Severity (SCAR‐S) [25]. However, it is important to note that these findings did not report on the statistical significance of their results [25]. Furthermore, two single‐blinded RCTs [21, 38] provided additional evidence supporting the efficacy of HA filler in this context. Both studies reported favorable outcomes, with the study by Siperstein et al. [21]. demonstrating statistically significant improvements in the Quantitative Global Scarring Grading System (QGSGS), GAIS, and the number of acne scars. Similarly, the NCT03127384 [38] trial found significant improvements in acne scarring as measured by GAIS, with 96% of participants showing improvement at the 1‐month follow‐up, which sustained at 83% at the 3‐month follow‐up. Notably, both RCTs reported that the HA filler intervention was well‐tolerated, with no serious adverse events, such as granulomas, being observed.

### 4.2 Combination Treatment With Hyaluronic Acid Filler

Subcision is a widely used surgical technique for managing acne scars [39]. It involves inserting a needle or blunt cannula beneath the scar and using a fanning, back‐and‐forth motion parallel to the skin surface to release fibrotic strands and elevate the atrophic scars [40]. The use of needles in this procedure is increasingly being replaced by blunt cannulas, which have shown superior efficacy and a better side effect profile [41].

The dual‐plane technique, commonly described in the literature for addressing aesthetic concerns and rejuvenation, has been adapted for acne scar treatment [13, 19]. In this context, the technique involves first filling the atrophic dermal component of the scar, followed by subcision and the placement of HA to address the subdermal component [13]. The study on this technique reported improvement in acne scarring after two sessions; however, it comes with caution as it lacked data or statistical analysis as well as objective assessment [13]. Furthermore, Mehrabi et al. [19] published a randomized, split‐face clinical trial of 82 patients who received the dual‐plane technique followed by two types of HA fillers: cross‐linked (CL‐HA) and non‐cross‐linked (NCL‐HA). The results demonstrated that CL‐HA achieved earlier aesthetic improvements compared to NCL‐HA, which were noticeable and sustained over the long term. Despite both fillers showing statistically significant improvements in the volume of atrophic scars after a minimum of three follow‐up visits, the numerical differences were minor, and patient satisfaction did not show a significant change between the initial and final visits [19]. In a smaller scale study, Dastgheib et al. [23] applied a similar technique in a randomized, single‐blind trial with 12 patients. While the study did not find statistically significant changes in scar depth, physician analysis, or clinical scoring, there was a significant reduction in boxcar scars [23]. Notably, the study reported statistically significant visual analog scale (VAS) scores, which could be attributed to the lack of subject blinding, as the objective analysis did not support these findings [23].

The triple step acne scar revision technique (TSART), described by Mohammed and Al‐Dhubaibi [22] as a new complementary modality for acne scar treatment, essentially mirrors the dual‐plane technique with minor variations. The study on TSART involved 82 patients who received either HA filler or no further intervention. Both groups showed statistically significant improvement in clinical efficacy, particularly in the treatment of rolling and boxcar scars, though not for icepick scars. Notably, the TSART group demonstrated a statistically significant improvement in patient satisfaction compared to the control group [22].

Ebrahim et al. [26] treated 40 patients with three subcision sessions at 4‐week intervals, followed by either HA filler or PLLA threads. Significant improvement was observed in 94.1% of patients treated with HA filler, 82.4% with PLLA threads, and 52.9% with subcision alone [26]. Rolling scars responded better than boxcar scars. Additionally, 76.5% of patients reached a grade 1 on the Goodman and Baron scale with HA filler, compared to 70.6% with threads, and none with subcision alone [26].

Behrangi et al. [20] investigated the combination of botulinum toxin A (BTA) and HA filler in the treatment of acne scars, comparing two groups that received BTA in conjunction with either CL‐HA or NCL‐HA. Both groups exhibited a statistically significant improvement in Goodman and Baron scores, though the numerical differences were modest, likely due to the small sample size [20]. Despite the observed improvements, patient satisfaction did not reach statistical significance, suggesting that the clinical impact of the treatment may be limited [20].

The use of lasers in treating acne scars is a common practice, although outcomes can vary. Abdelwahab, Omar, and Hamdino [27] found that combining HA fillers with subcision resulted in statistically and clinically significant improvements compared to subcision alone. While fractional CO_2_ laser (FACL) treatment also led to slightly better outcomes than subcision alone, there was no significant difference observed between the HA filler and FACL groups [27]. Similarly, a study by Mahamoud et al. [42] demonstrated that combining a fractional CO_2_ laser with NCL‐HA yielded statistically significant improvements in both quantitative and qualitative Goodman and Baron scores. The same study also explored the use of platelet‐rich plasma (PRP) in combination with FACL, finding slightly higher results, although the difference between the two treatment modalities was not statistically significant [42]. Furthermore, in a retrospective case series by Akerman et al. [24], the combination of a non‐ablative fractional laser (NAFL) with NCL‐HA led to modest improvements in acne scarring, with no adverse events reported.

In a case series by Belmontesi [14], trifarotene, a fourth‐generation retinoid, was utilized as short‐contact therapy for 4 months, followed by 3–10 injections of an HA skin booster filler into the scars. The study reported significant clinical improvement, with some patients experiencing nearly complete resolution of atrophic acne scars [14]. Interestingly, a phase 4 clinical trial comparing trifarotene to a vehicle control demonstrated a significant reduction in atrophic acne scars, as measured by the subjective global assessment (SGA) and investigator global assessment (IGA) scales [43]. However, the lack of additional studies using this combination, along with the absence of a control group in Belmontesi's [14] study, makes it difficult to determine whether HA injections provided a synergistic effect.

### 4.3 Poly‐L Lactic Acid

Two studies evaluated PLLA monotherapy in treating acne scars, both demonstrating a significant reduction in scarring [28, 29]. Beer [29] conducted a study with seven treatment sessions, while Sapra et al. [28] reported on a regimen of three to four sessions; both studies yielded similar positive outcomes. Various injection techniques, including depot, tunneling, and fanning, were employed. Notably, Sapra et al. [28] utilized a larger volume of PLLA for depot and tunneling techniques, potentially reflecting the varying severity of acne scarring among individual patients. Adverse events were reported by Sapra et al. [28] including a palpable, non‐visible nodule, acne, and post‐inflammatory hyperpigmentation, whereas Beer [29] reported no treatment‐emergent adverse events.

### 4.4 Calcium Hydroxyapatite

CaHA was investigated in four studies, each involving a single treatment session. One study assessed CaHA as a monotherapy, administering a single session to all patients, with additional treatment offered during follow‐up if necessary [17]. Improvement was observed 12 months post‐treatment; however, several adverse effects were reported, including extrusion, nodule formation, and further scar development [17].

The remaining studies evaluated the combination of CaHA with energy‐based devices (EBDs) [15] or ultrasound‐based interventions, such as high‐intensity focused ultrasound (HIFU) [18] and microfocused ultrasound with visualization (MFU‐V; Ultherapy) [16]. The retrospective study by Koren et al. [15] involving 352 patients compared outcomes among those treated with EBDs alone, CaHA alone, or a combination of both. The study found that FACL was more effective for treating acne scars than CaHA alone, though combining these two modalities resulted in a statistically significant improvement [15]. In contrast, the studies exploring ultrasound‐based therapies revealed that the combination with CaHA did not provide a synergistic benefit over CaHA alone [16].

### 4.5 Polymethylmethacrylate

A pilot study by Joseph et al. [10] investigating PMMA for acne scarring reported impressive safety and efficacy results. However, caution is warranted due to several concerns highlighted by Behshad [44], including potential bias, methodological issues, a short follow‐up period, and data omission. Nevertheless, two RCTs assessed the efficacy of PMMA for acne scarring [31, 33]. Both trials demonstrated statistically significant favorable outcomes as measured by the Acne Scar Rating Scale (ASRS), Physician and Subject Global Aesthetic Improvement Scale (PGAIS and SGAIS), with no treatment‐related adverse events reported [31, 33]. Additionally, two retrospective chart reviews examined the use of PMMA in treating depressed acne scars [30, 32]. Both reviews found that the majority of patients experienced significant improvement and reported high levels of satisfaction [30, 32]. However, none of the two studies indicated specific measures used to evaluate the patients.

### 4.6 Polycaprolactone

PCL is a novel collagen stimulator that offers immediate volumizing effects due to the microspheres in which it is suspended, and it maintains this volume through sustained collagen production triggered by the healing cascade [11]. It appears to have a longer lasting effect compared to PLLA [11]. An Iranian pilot study involving 10 participants explored the use of PCL in combination with radiofrequency (RF)‐assisted subcision for treating acne scars [34]. The study reported significant improvement in the appearance of acne scars, with notable reductions across all scar types, including icepick, boxcar, and rolling scars [34]. Reported side effects included localized edema, ecchymosis, and a burning sensation, which the authors attributed to RF‐assisted subcision rather than being inherent to PCL and were described as mild [34]. The study did not include a comparison group receiving RF‐assisted subcision alone, limiting the conclusions that can be drawn. As such, while the combination of PCL with RF‐assisted subcision appears to be safe and potentially effective, further research is needed to establish its efficacy relative to other treatments.

### 4.7 Techniques

Out of all the included fillers studies reviewed, 11 used the filler as monotherapy. While seven of the included studies used the fillers combined with Subcision technique, most of these studies range from 2019 to 2024. Two of the CaHA studies used Radiesse on 362 subjects in combined with Fractional CO_2_ Laser, non‐ablative laser, and microfocused ultrasound with visualization. In contrast, 10 out of 13 HA filler studies used it with either Subcision or with others such as Botulinum Toxin A, Fractional CO_2_ Laser, non‐ablative laser, and Topical retinoid. Regarding the PMMA filler, one study done in USA used a different method than all the included studies, they used microneedling on 44 subjects followed by subcision. While the one study on PCL filler was used with subcision technique. In conclusion, subcision is a widely used surgical technique for managing acne scars [39]. Subcision with dermal filler has fewer side effects with immediate results compared with other techniques.

### 4.8 Safety and Tolerability

Across all fillers reviewed, safety remains a critical consideration. Besides PMMA (Bellafill), none of the injected fillers are FDA approved in treating acne scars. Nevertheless, HA has the most robust efficiency and safety record, with common side effects being localized and transient [45]. PLLA and CaHa PCL are well‐tolerated, though prolonged edema has been noted in cases involving higher injected volumes [46]. Among the fillers, PMMA raises the most safety concerns due to its permanent nature, though adverse events like granulomas remain rare [47].

While HA remains a safe and reliable option due to its well‐established efficacy and safety record [45], CaHA and PLLA also present strong potential as they are well‐tolerated and their ability to promote sustained collagen stimulation. However, it is advisable to combine fillers with other modalities such as EBDs, subcision, or chemical peels to synergize outcomes. In terms of safety, PCL is comparable to other treatments, with common side effects including localized edema and ecchymosis reported in the included studies. In a retrospective study of 780 patients, prolonged edema was associated with higher injected volumes [46]. Specifically, patients with prolonged edema had an average injected volume of 8.36 mL, compared to 4.88 mL in those without prolonged edema [46]. Nevertheless, it does not appear that there is specific safety concerns associated with PCL, making it a well‐tolerated option [46]. Among the fillers, HA has the most robust evidence supporting its use for acne scars, followed by PMMA. However, PMMA raises the most concerns due to its status as a permanent filler and the higher incidence of reported side effects, such as granuloma formation, although these side effects remain rare overall [47].

### 4.9 Limitations and Future Research

The literature is notably deficient in high‐quality RCTs evaluating the use of the commercially available dermal fillers for acne scars. It is important to note that most studies included in this review were of low quality, as indicated by their scores on quality assessments and small sample sizes. As with any study, this review has its limitations, including the heterogeneity of the included studies, particularly due to the lack of high‐quality RCTs. Additionally, many of the studies reviewed had small sample sizes, which limits the generalizability of the findings. To address these limitations, future research should focus on conducting randomized, controlled, split‐face studies with an adequate number of participants and a detailed examination of different scar subtypes.

## Author Contributions

The author confirms sole responsibility for the following: study conception and design, data collection, analysis and interpretation of results, and manuscript preparation.

## Ethics Statement

The author confirm that the ethical policies of the journal, as noted on the journal's author guidelines page, have been adhered to. No ethical approval was required as this is a review article with no original research data.

## Conflicts of Interest

The author declares no conflicts of interest.

## Data Availability

The data that support the findings of this study are available from the corresponding author upon reasonable request.
